# *QuickStats:* Percentage* of Adults Aged ≥25 Years^†^ Who Met the 2018 Federal Physical Activity Guidelines for Both Muscle-Strengthening and Aerobic Physical Activity,^§^ by Educational Attainment — United States, 2022

**DOI:** 10.15585/mmwr.mm7322a3

**Published:** 2024-06-06

**Figure:**
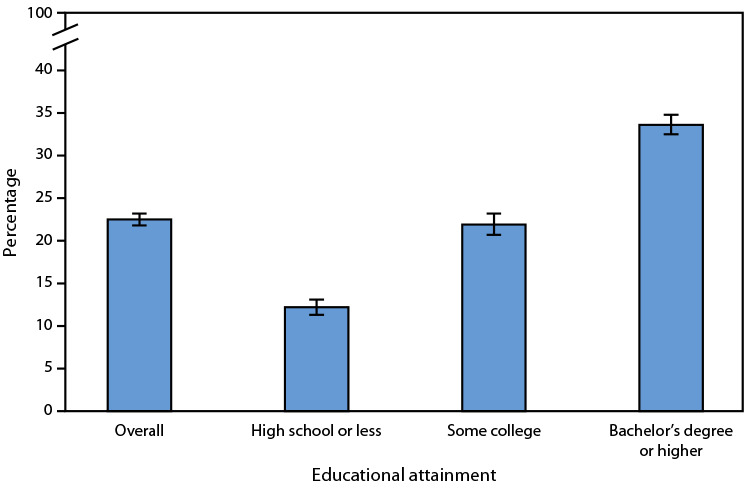
In 2022, 22.5% of adults met federal guidelines for both muscle-strengthening and aerobic physical activity. The percentage of adults who met these guidelines increased with increasing educational attainment, from 12.2% among adults who completed high school or less to 33.6% among those with a bachelor’s degree or higher.

**Supplementary Table:** https://stacks.cdc.gov/view/cdc/155046

**Source:** National Center for Health Statistics, National Health Interview Survey, 2022. https://www.cdc.gov/nchs/nhis.htm

For more information on this topic, CDC recommends the following link: https://www.cdc.gov/physical-activity-basics/benefits/index.html

